# Growth patterns of young achondroplasia patients in Korea and predictability of neurosurgical procedures

**DOI:** 10.1186/s13023-023-02929-6

**Published:** 2023-10-05

**Authors:** Jong Seok Lee, Youngbo Shim, Tae-Joon Cho, Seung-Ki Kim, Jung Min Ko, Ji Hoon Phi

**Affiliations:** 1 Division of Pediatric Neurosurgery, Seoul National University Children’s Hospital, Seoul National University College of Medicine, 101 Daehak-ro, Jongno-gu, Seoul, 03080 Republic of Korea; 2 Division of Pediatric Orthopedics, Seoul National University Children’s Hospital, Seoul National University College of Medicine, Seoul, Republic of Korea; 3 Department of Pediatrics, Seoul National University Children’s Hospital, Seoul National University College of Medicine, 101 Daehak-ro, Jongno-gu, Seoul, 03080 Republic of Korea

**Keywords:** Achondroplasia, Head circumference, Growth, Hydrocephalus, Foramen magnum decompression, Ventriculoperitoneal shunt

## Abstract

**Background:**

Achondroplasia is an autosomal dominant disorder mainly affecting bony growth, typically resulting in markedly short stature. From a neurosurgical viewpoint, patients sometimes develop spinal cord compression at the narrowed foramen magnum and hydrocephalus. This study aims to construct growth references for height, weight, and head circumference (HC) of young achondroplasia patients in Korea and to evaluate the predictability of the necessity and timing of neurosurgical procedures through growth patterns.

**Methods:**

Growth data were collected from achondroplasia patients who visited our institution between January 2002 and August 2022. First, we constructed percentile growth curves of height, weight, and HC for the patients under 3 years of age with the generalized additive model for location, scale, and shape (GAMLSS). Second, the growth patterns of the patients with hydrocephalus who underwent neurosurgical procedures such as foramen magnum decompression (FMD) and ventriculoperitoneal (VP) shunt were analyzed.

**Results:**

There were 125 achondroplasia patients, including 67 males and 58 females. Among 125 patients, 46 underwent FMD, and 5 underwent VP shunt. As short stature and macrocephaly were typical characteristics of achondroplasia, the height of achondroplasia was lower than that of the general population, and HC in achondroplasia showed accelerated growth postnatally. There were no significant changes in HC in hydrocephalus patients before they underwent neurosurgical procedures. The influence of hydrocephalus on the growth patterns of HC in achondroplasia seemed insignificant.

**Conclusion:**

Growth references for height, weight, and HC in young achondroplasia patients were constructed. It is the first report of growth patterns of achondroplasia in Korea. Unlike other pediatric patients, the diagnosis of hydrocephalus and the necessity of neurosurgical procedures are hard to be predicted with HC in achondroplasia. Neuroimaging should be considered for achondroplasia patients with neurological symptoms.

## Background

Achondroplasia is the most common skeletal dysplasia affecting bony growth that is typically characterized by markedly short stature [1, 2]. It is an autosomal dominant disorder caused by an activating mutation of fibroblast growth factor receptor type 3 (FGFR 3) [2–5]. The incidence of achondroplasia is one of 25,000–30,000 live births, which makes it a rare disease [1, 2, 5–7]. From a neurosurgical viewpoint, achondroplasia patients possess several risks for neurological deterioration [5, 8–10]. One major problem is spinal cord injury caused by the narrow skull base and foramen magnum. Young infants can develop sudden apneic death due to compression of the high cervical cord [1, 5, 6]. Clinical surveillance and application of foramen magnum decompression (FMD) is the solution to this problem [1, 5, 11–13]. The other serious trouble is the development of hydrocephalus [14]. Ventricular enlargement with thinning of the cerebral mantle is often observed with progressively increased head circumference (HC). However, macrocephaly is a universal feature of achondroplasia and can be presented without overt hydrocephalus [1, 15]. Furthermore, the symptoms of hydrocephalus in infants with achondroplasia are often ambiguous. Lethargy and irritability, common symptoms of infantile hydrocephalus, are rare in achondroplasia, and patients usually exhibit insidious developmental delay due to thinning of the cerebral mantle [1, 16]. The proportion of hydrocephalus requiring shunting in achondroplasia patients is known to be about 4%, but recent studies report a much lower incidence of symptomatic, overt hydrocephalus [5, 9, 16]. Therefore, diagnosing and appropriately managing hydrocephalus in infants with achondroplasia is a difficult task for many clinicians.

We tried to evaluate the relationship between changes in HC and the likelihood of ventriculoperitoneal (VP) shunt operation in achondroplasia patients because, in general, intracranial hypertension can cause acceleration of head growth in young patients with hydrocephalus [17]. However, there was no reference growth data including HC for achondroplasia patients in Korea. Therefore, we aimed to construct growth references of height, weight, and HC of young achondroplasia patients in Korea. Using the reference data, we ultimately evaluated the predictability of the necessity and timing of neurosurgical procedures through growth patterns.

## Materials and methods

### Study design and population

The study was approved by the Institutional Review Board of the author’s institution (IRB no. 2211-064-1377). Informed consent was waived due to the retrospective design.

**Fig. 1:**
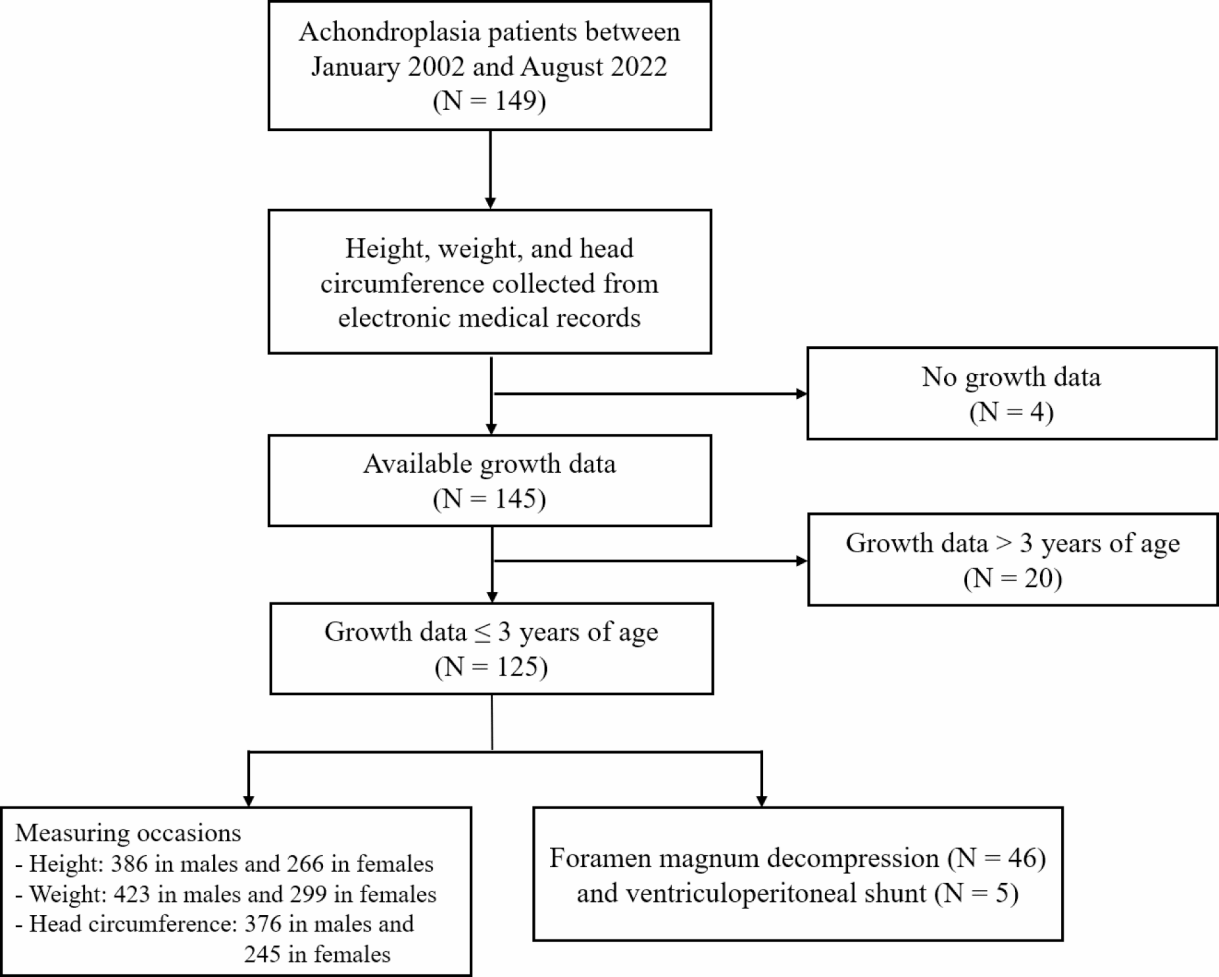
Flowcharts of the patients

One hundred forty-nine achondroplasia patients visited our institution between January 2002 and August 2022. Growth data of height, weight, and HC were measured when patients visited outpatient clinics or were admitted to wards. The data were collected from electronic medical records. We included growth data of 0–3 years of age because the HC rapidly increases in 0–3 years and reaches nearly 90% of adults in the general population [18]. The Korea Disease Control and Prevention Agency in 2017 [19] and the National Center for Health Statistics in 2000 [20] provided HC growth curves of the standard pediatric population only under 3 years of age. Also considered is that achondroplasia patients over 3 years old largely escape the vulnerable periods for neurosurgical procedures such as FMD and VP shunt if patients were examined from early infancy [16]. We excluded 4 patients with no growth data and 20 patients with only growth data over 3 years of age. Finally, the growth data of 125 patients were included in the study (Fig. 1).

### Criteria for the treatment of FMD and VP shunt

Surgical treatments were decided based on the clinical presentations and magnetic resonance imaging (MRI) findings at the first visit and follow-up. Cervical myelopathy was diagnosed with overt neurological abnormalities, a history of respiratory depression or arrest, or evidence of spinal cord injury on MRI (high signal intensity on T2-weighted images). We performed FMD on patients with cervical myelopathy and patients with severe stenosis of the cervical spinal cord (indentation of the cord greater than 25% of the normal cord thickness on MRI) [13, 16, 21–23]. It had been considered that severe stenosis of the cervical spinal cord was an indentation of the cord greater than 50% of the normal cord thickness before the literature was published in 2021 [16].

Hydrocephalus was diagnosed when there were definite neurological symptoms with corresponding imaging findings. Neurological abnormalities included bulged and tense fontanelle, scalp vein engorgement, and limited eyeball movement or sunset sign. However, some patients with hydrocephalus showed vague symptoms like poor oral intake, irritability, or simple developmental delay. Papilledema on the fundoscopic exam or characteristic imaging findings were important in those cases. Imaging findings included progressively increasing ventricular size on serial images or overtly enlarged ventricles with periventricular T2 high signal intensity on MRI. A previous study by ours showed that “frontal horn width” and “Evan’s ratio” were significant predictors for hydrocephalus, and they came into consideration for the diagnosis of hydrocephalus [16].

The treatment of hydrocephalus in achondroplasia was individualized and varied according to the clinical situation. If the patient had increasing ventricular volume and concomitant stenosis at the foramen magnum that met the indication of FMD, FMD was performed first. For some patients who presented both with hydrocephalus and cervical myelopathy, hydrocephalus was relieved by FMD [5, 24]. VP shunt was performed if initial FMD did not reverse the progressive ventriculomegaly and associated symptoms or if there was no craniovertebral stenosis necessitating FMD. Endoscopic third ventriculostomy (ETV) was recently considered a treatment option for some patients requiring VP shunt [7, 14, 25, 26].

### Statistical analysis

Growth curves and percentile graphs of height, weight, and HC under 3 years of age were constructed using the generalized additive model for location, scale, and shape (GAMLSS) model in R. We retrospectively compared differences in the mean values of height, weight, and HC at birth, 1 year, 2 years, and 3 years of age between achondroplasia patients and the general population using independent T-tests and Mann-Whitney tests by IBM SPSS® Statistics 27.0 (2017 IBM Corp. NY, USA). We compared our growth data with the American growth data presented by the American academy of pediatrics as global guidelines for achondroplasia [3]. We also compared HC at birth, 1 year, 2 years, and 3 years of age according to the presence of hydrocephalus using independent T-tests and Mann-Whitney tests. A *p*-value less than 0.05 was considered statistically significant.

## Results

### Patients’ characteristics

Among 125 achondroplasia patients, there were 67 males and 58 females. The range of age at diagnosis was between 3 months and 3 years. Hydrocephalus was diagnosed in 15 patients (12.0%) during the follow-up. FMD was performed in 46 patients (36.8%), VP shunt was performed in 5 patients (4%), and ETV was performed in 2 patients (1.6%). For constructing growth curves of height, weight, and HC, only values under the age of 3 were used. Measuring occasions for height were 386 for males, 266 for females, and a total of 652. Measuring occasions for weight were 423 for males, 299 for females, and a total of 722. Measuring occasions for HC were 376 for males, 245 for females, and a total of 621.

### Growth curves of height

**Fig. 2:**
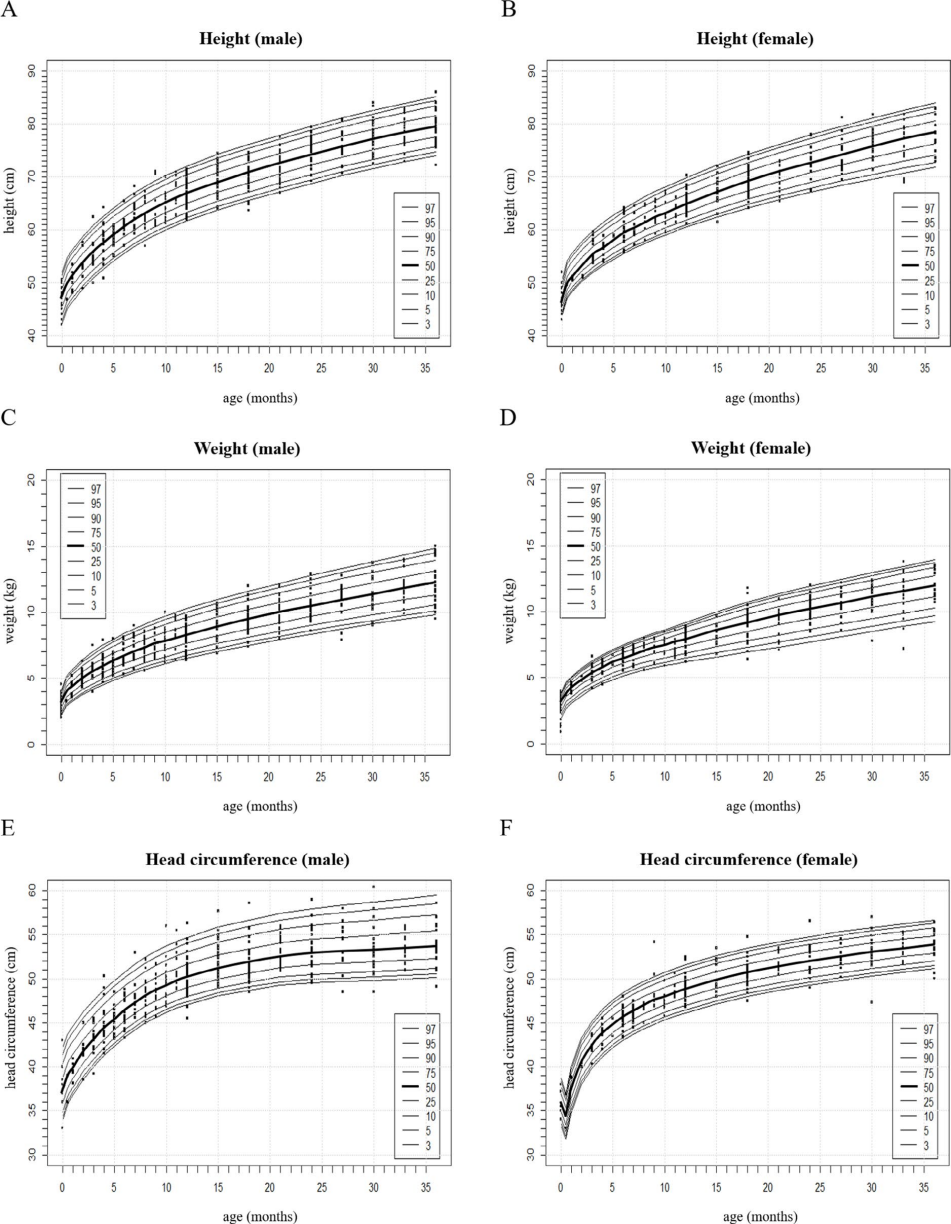
Scatter plots and percentile growth curves of height for **(A)** males and **(B)** females, weight for **(C)** males and **(D)** females, and head circumference for **(E)** males and **(F)** females in achondroplasia. The x-axis represents the age of months, and the y-axis represents the height **(A, B)**, weight **(C, D)**, and head circumference **(E, F)**. The category shows the percentage. There is no difference in the curves of head circumference between whole achondroplasia patients **(E, F)** and patients without hydrocephalus (data not presented)

Scatter plots and percentile growth curves of height were constructed for male and female achondroplasia patients (Fig. 2-A, B). The x-axis is marked from 0 to 36 months at 1-month intervals, and the y-axis is marked from 40 cm to 1 cm intervals. The category shows the percentage. The mean values of the height in Korean achondroplasia patients at birth, 1 year, 2 years, and 3 years of age were compared with those of American achondroplasia patients presented by the American academy of pediatrics [3, 27] and the Korean general population presented by the Korea Disease Control and Prevention Agency in 2017 [19] (Table 1).

**Table 1:** The mean height, weight, and head circumference of Korean achondroplasia patients, American achondroplasia patients, and the Korean general population at birth, 1 year, 2 years, and 3 years of age. The values shown in parentheses mean how the mean growth value of Korean achondroplasia deviated from that of the reference population

	Korean achondroplasia (n = 125)		American achondroplasia [3, 27, 28]		Korean general population [19]	
	**Male (n = 67)**	**Female (n = 58)**	**Male**	**Female**	**Male**	**Female**
Height, cm						
Birth	47.2 (-2 ~ 0 SD_A, -2 ~ -1 SD_K)	46.7 (-2 ~ 0 SD_A, -2 ~ -1 SD_K)	50	50	49.9	49.1
1 year	67.0 (> 2 SD_A, < -3 SD_K)	65.3 (> 2 SD_A, < -3 SD_K)	62	60	75.7	74.0
2 years	75.0 (2 SD_A, < -3 SD_K)	73.3 (2 SD_A, < -3 SD_K)	71	69	87.1	85.7
3 years	79.2 (0 ~ 2 SD_A, < -3 SD_K)	77.7 (0 ~ 2 SD_A, < -3 SD_K)	77	76	96.5	95.4
Weight, kg						
Birth	3.2 (-2 ~ 0 SD_A, -1 ~ 0 SD_K)	3.0 (-2 ~ 0 SD_A, -1 ~ 0 SD_K)	3.4	3.4	3.3	3.2
1 year	8.3 (0 ~ 2 SD_A, -2 ~ -1 SD_K)	7.9 (-2 ~ 0 SD_A, -1 SD_K)	8.2	8.2	9.6	8.9
2 years	10.7 (0 ~ 2 SD_A, -2 ~ -1 SD_K)	10.4 (0 ~ 2 SD_A, -1 ~ -0 SD_K)	10.1	10.1	12.2	11.5
3 years	12.2 (-2 ~ 0 SD_A, -2 ~ -1 SD_K)	12.0 (-2 ~ 0 SD_A, -2 ~ -1 SD_K)	12.4	12.3	14.7	14.2
Head circumference, cm						
Birth	37.9 (-2 ~ 0 SD_A, 2 ~ 3 SD_K)	35.9 (-2 ~ 0 SD_A, 1 ~ 2 SD_K)	39	37.5	34.5	33.9
1 year	50.0 (-2 ~ 0 SD_A, > 3 SD_K)	49.1 (-2 ~ 0 SD_A, > 3 SD_K)	50.5	49.5	46.1	44.9
2 years	53.1 (-2 ~ 0 SD_A, > 3 SD_K)	51.9 (-2 ~ 0 SD_A, > 3 SD_K)	54.5	54	48.3	47.2
3 years	54.2 (-2 ~ 0 SD_A, 2 ~ 3 SD_K)	53.8 (-2 ~ 0 SD_A, > 3 SD_K)	55	54.5	49.8	48.8

*SD* Standard deviation, *SD_A* SD of American achondroplasia patients, *SD_K* SD of Korean general population

At birth, the height of Korean achondroplasia was lower than that of the Korean general population, but it was still above the − 2 standard deviation (SD) of the general population. At 1 year, the height of achondroplasia already fell below − 3 SD of the height of the general population. The mean height of 2-year-old achondroplasia patients was lower than that of the general population at 1 year of age. The growth velocity rapidly decreased in the first year of life in achondroplasia, and the height difference between the two groups increased as they grew older until the age of 3.

The Korean achondroplasia patients showed a sharper increase in height between 0 and 2 years of age compared with the American achondroplasia patients. The height of the Korean and American achondroplasia patients became similar again at the age of 3 (within 2 SD).

### Growth curves of weight

Scatter plots and percentile growth curves of weight were constructed for male and female achondroplasia patients (Fig. 2-C, D). The x-axis is marked from 0 to 36 months at 1-month intervals, and the y-axis is marked from 0 kg at 1-kg intervals. The mean values of the weight of achondroplasia patients were compared with those of American achondroplasia patients presented by the American academy of pediatrics [3, 27] and the Korean general population presented by the Korea Disease Control and Prevention Agency in 2017 [19] (Table 1).

The mean weight of Korean achondroplasia patients was also lower than that of the Korean general population, but it was always above − 2 SD of the weight of the general population. Also, there were no significant differences in the weight between the Korean and American achondroplasia patients.

### Growth curves of HC

Scatter plots and percentile growth curves of HC were constructed for male and female achondroplasia patients (Fig. 2-E, F). The x-axis is marked from 0 to 36 months at 1-month intervals, and the y-axis is marked from 30 cm to 1 cm intervals. The mean values of the HC of achondroplasia patients were compared with those of American achondroplasia patients presented by the American academy of pediatrics [3, 28] and the Korean general population presented by the Korea Disease Control and Prevention Agency in 2017 [19] (Table 1).

The mean HC of Korean achondroplasia patients is larger than that of the Korean general population, as macrocephaly is one of the typical features of achondroplasia patients. At birth, it appears to be around + 2 SD (male: 2–3 SD, female: 1–2 SD), and it became larger than + 3 SD of the general population at the age of 1. It is constantly higher than + 2 SD of the general population. It showed accelerated head growth postnatally compared with the general population. The HC of the Korean achondroplasia patients was lower than that of the American achondroplasia patients, but there were no statistically significant differences.

Growth curves of HC were also constructed for achondroplasia patients without hydrocephalus. However, there was no difference in the curves of head circumference between whole achondroplasia patients (Fig. 2-E, F) and patients without hydrocephalus (data not presented).

### Growth patterns of patients with hydrocephalus

There were 15 patients diagnosed with presumptive hydrocephalus. Among them, 11 were males, and 4 were females. Patients underwent either FMD (n = 13, male:female = 9:4) or VP shunt (n = 5, male:female = 3:2). Both were performed on 3 patients.

**Fig. 3:**
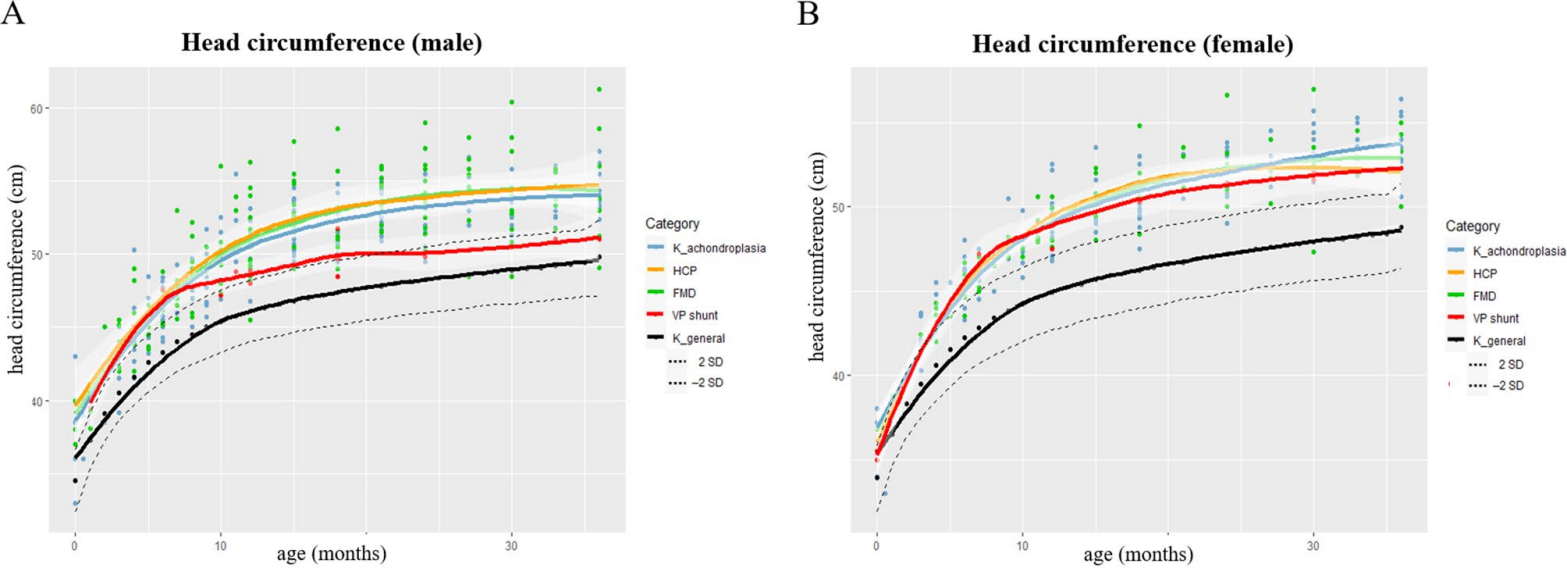
Scatter plots and growth curves of head circumference (HC) for **(A)** male and **(B)** female achondroplasia patients. The x-axis represents the age of months, and the y-axis represents the head circumference. Growth curves show the mean value of HC with 95% confidence intervals in whitish areas. The category represents Korean achondroplasia patients (K_achondroplasia), patients with hydrocephalus (HCP), and patients who underwent foramen magnum decompression (FMD) or ventriculoperitoneal (VP) shunt among HCP with corresponding colors. HC of the Korean general population (K_general) is shown in black lines with ± 2 standard deviation (SD) values in dotted lines. Among patients with HCP, three male patients underwent VP shunt at 5, 6, and 19 months of age, and two female patients underwent VP shunt at 35 and 36 months of age

We compared HC between patients with and without hydrocephalus. We also analyzed the HC according to what neurosurgical procedures the patients underwent. Scatter plots and growth curves of HC were constructed for whole achondroplasia patients, patients with hydrocephalus, and patients who underwent FMD or VP shunt (Fig. 3). Growth curves represented the mean HC with 95% confidence intervals in whitish areas. There seemed similar growth curves with overlapping 95% confidence intervals between 4 categorical groups, both in males and females. Only the growth curve of VP shunt after 6–7 months of age in males showed a different pattern. Among patients with hydrocephalus, three male patients underwent VP shunt at 5, 6, and 19 months of age, and two female patients underwent VP shunt at 35 and 36 months of age. The growth velocity of HC decelerated after two male patients underwent VP shunt each at 5 and 6 months of age. There were no growth spurts of HC before the operation, and the value of HC was not high in patients who underwent VP shunt compared to that of whole patients. We also compared the mean values of HC between patients with and without hydrocephalus at birth, 1 year, 2 years, and 3 years of age (Table 2). There were no statistically significant differences for each age in both males and females.

**Table 2:** Differences in head circumference at birth, 1 year, 2 years, and 3 years of age between achondroplasia patients with and without hydrocephalus. The values represent the mean head circumference (cm)

	Male (n = 67)			Female (n = 58)		
	**HCP** **(n = 11)**	**No HCP** **(n = 56)**	***p*** **-value**	**HCP** **(n = 4)**	**No HCP** **(n = 54)**	***p*** **-value**
Birth	38	37.9	1.000	35.5	36.1	1.000
1 year	51.3	49.7	0.230	49.8	49.0	0.373
2 years	53.5	53.0	0.679	51.4	51.9	0.752
3 years	55.4	54.0	0.491	52.3	53.8	0.375

*HCP* Hydrocephalus

**Fig. 4:**
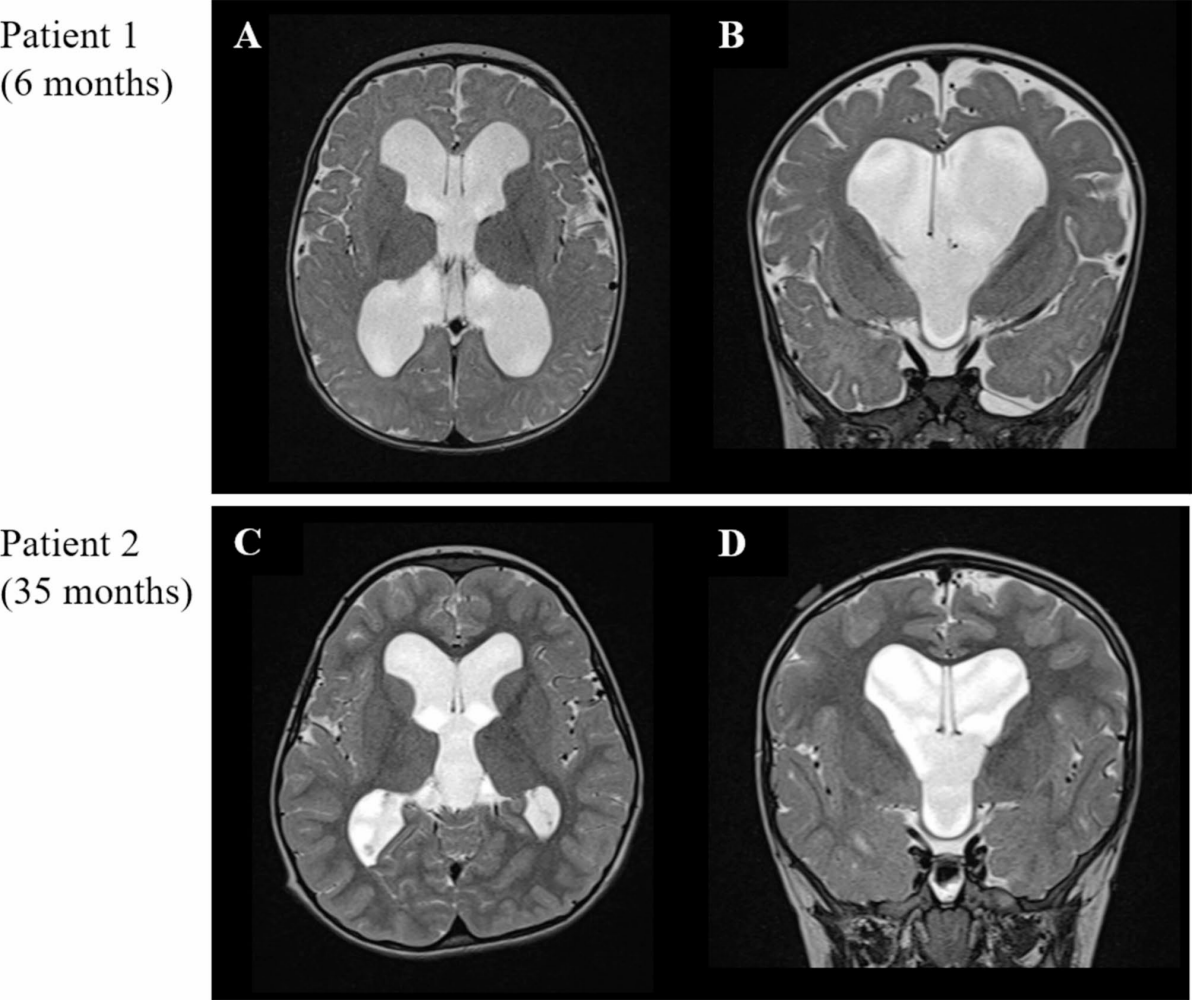
Brain magnetic resonance imaging (MRI) was taken just before the operation in two achondroplasia patients who underwent ventriculoperitoneal shunt. Each was performed at 6 months (patient 1, **A**, **B**) and 35 months (patient 2, **C**, **D**) of age. Both patients showed marked dilatation of lateral and third ventricles on T2-axial (**A**, **C**) and T2-coronal (**B**, **D**) images. (**A**, **B**) Frontal horn width = 67.85 mm, Evan’s ratio = 0.51 (**C**, **D**) Frontal horn width = 61.31 mm, Evan’s ratio = 0.42

**Fig. 5:**
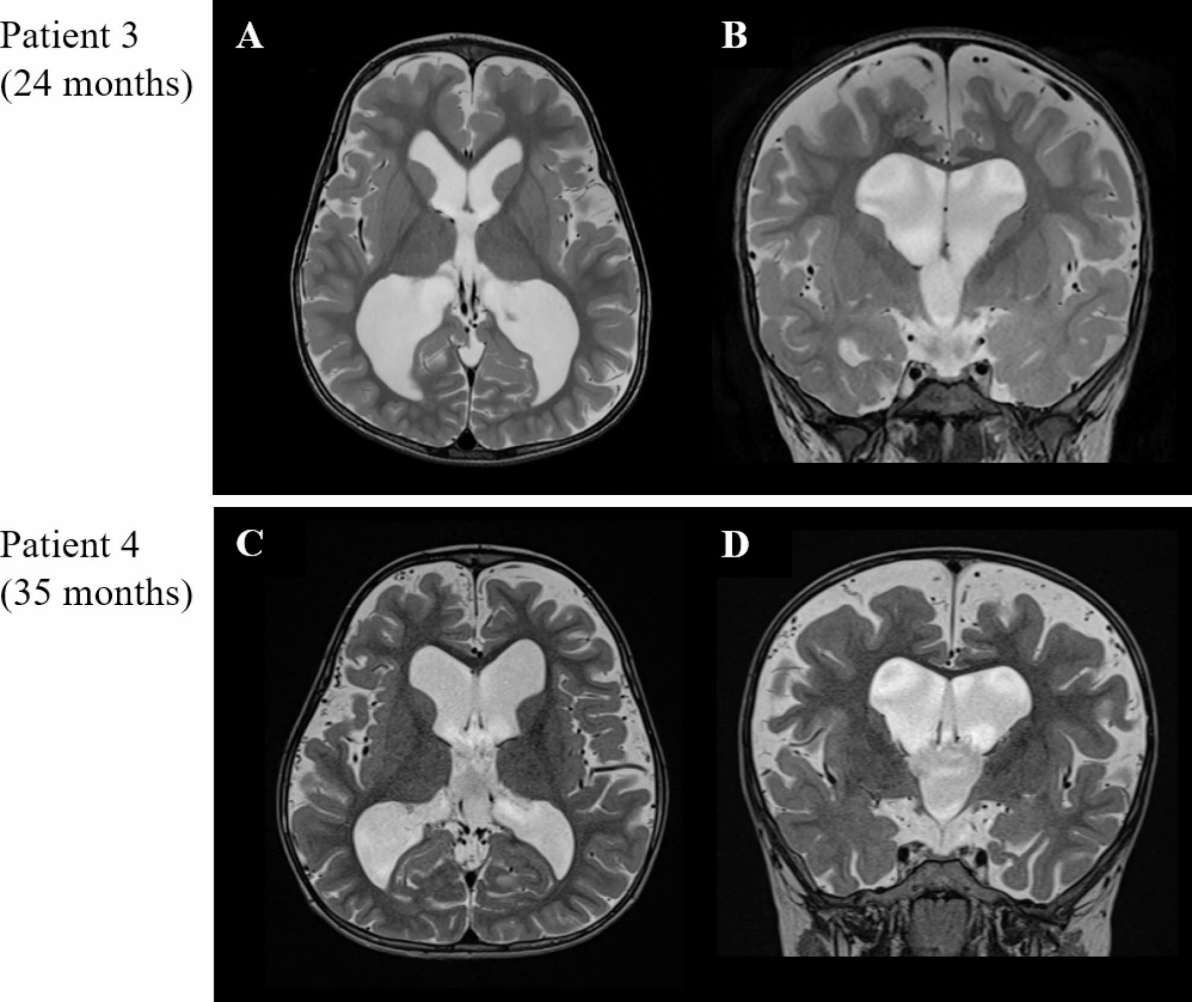
Brain magnetic resonance imaging (MRI) was taken in two achondroplasia patients who didn’t undergo ventriculoperitoneal shunt. Each was performed at 24 months (patient 3, **A**, **B**) and 35 months (patient 4, **C**, **D**) of age. Both patients showed mild to moderate dilatation of lateral and third ventricles with excessive cerebrospinal fluid volume in the cortical subarachnoid space on T2-axial (**A**, **C**) and T2-coronal (**B**, **D**) images. (**A**, **B**) Frontal horn width = 57.58 mm, Evan’s ratio = 0.36 **(C, D)** Frontal horn width = 67.03 mm, Evan’s ratio = 0.39

Brain MRI of the two patients who underwent VP shunt was shown (Fig. 4). Both patients presented developmental delay without overt neurological changes. One patient showed hydrocephalus without cervical myelopathy, and the other patient showed serial dilation of ventricles on MRI after FMD was performed. Two patients underwent VP shunt at 6 and 35 months of age. Markedly dilated ventricles were presented on imaging taken just before the operation. In patient 1 (Fig. 4-A, B), the frontal horn width was 67.85 mm, and Evan’s ratio was 0.51. In patient 2 (Fig. 4-C, D), the frontal horn width was 61.31 mm, and Evan’s ratio was 0.42. On the other hand, brain MRI of achondroplasia patients who didn’t undergo VP shunt was shown (Fig. 5). Brain MRI was taken each at 24 and 35 months of age. They also showed enlarged ventricles with excessive cerebrospinal fluid (CSF) volume in the cortical subarachnoid space and enlarged HC. However, they didn’t undergo VP shunt operations because they had no symptoms, and there was no further dilatation of ventricles on serial follow-up images. In patient 3 (Fig. 5-A, B), the frontal horn width was 57.58 mm, and Evan’s ratio was 0.36. In patient 4 (Fig. 5-C, D), the frontal horn width was 67.03 mm, and Evan’s ratio was 0.39. It seemed difficult to decide on whether surgical intervention should be performed or not merely based on the fragmented MRI imaging and changes in HC.

## Patients with hydrocephalus treated with ETV

The growth patterns of patients who underwent ETV were not analyzed in this study because only 2 patients underwent ETV. For both patients (one male and one female) who co-presented cervical myelopathy and hydrocephalus, FMD was first performed. They presented progressive hydrocephalus, and triventricular hydrocephalus was shown on MRI. They underwent ETV instead of VP shunt, and hydrocephalus was resolved in both patients. One patient was followed up for 8 months, and the other patient was followed up for 2 years and 9 months without evidence of hydrocephalus.

## Discussion

Growth patterns of height, weight, and HC in achondroplasia patients are quite different from those in the general population [27, 29–32]. Because enchondral bony growth is inhibited in achondroplasia, short stature and macrocephaly are characteristic features of achondroplasia patients [1, 2, 9]. Macrocephaly is known to be caused by increased brain volume with increased CSF in the subarachnoid space and enlarged cerebral ventricles [33]. However, there have been limited reports of growth in achondroplasia due to its rareness. Most previous reports on achondroplasia presented the natural course of the disease and the treatment strategies [3, 4, 10, 21, 27]. Growth in achondroplasia was described by several nations including the United States, Europe, Japan, Argentina, and Australia [4, 28–31, 34, 35]. Asian data was described by Japan, but the literature was not available anymore in the online database [31]. This is the first literature presenting growth data of height, weight, and HC in young achondroplasia in Korea, and it can also be the first updated Asian reference.

We additionally evaluated the impact of hydrocephalus and neurosurgical procedures on the HC in achondroplasia. Hydrocephalus and cervical myelopathy are critical diseases in the field of neurosurgery, which can cause irreversible neurological impairment or even death. However, there are some difficult points in diagnosis and deciding on the treatments for hydrocephalus, especially for patients with achondroplasia. Symptoms can be ambiguous, and ventriculomegaly without intracranial hypertension is prevalent in achondroplasia. On the other hand, there have been efforts to predict hydrocephalus with changes in the HC as a screening evaluation [17]. HC measurement is a quick, inexpensive, and non-invasive parameter. We also tried to evaluate patterns of HC as a screening parameter for predicting the necessity and the timing of the neurosurgical procedures on achondroplasia patients with hydrocephalus.

### Growth patterns of young achondroplasia patients

As short stature and macrocephaly were typical characteristics of achondroplasia, corresponding growth curves of height and HC were constructed. The mean weight in achondroplasia was constantly lower than in the general population. However, the difference was statistically insignificant. There could be a tendency for short patients to have low body weight, but it was not purely affected by height. Head size, body proportion, and body composition could also make differences in weight.

Growth patterns of Korean achondroplasia patients seemed similar to that of the American achondroplasia patients presented by the American academy of pediatrics [3, 27, 28]. There were only significant differences in the height between the two groups at 1 and 2 years of age. They became insignificant at 3 years of age. These differences could be due to ethnic differences or time differences that the American growth data of height was presented in 2008. Growth patterns of achondroplasia in our study also seemed similar to those in the European cohort study described by Merker et al. [31]. In this cohort study, the HC of achondroplasia was relatively normal during the first months of life, and growth velocity abruptly slowed down. It caused a significant decrease in height position on the general population growth curve approaching − 4.0 SD before 1 year of age and − 5.0 SD at 2 years of age. Also, HC accelerated postnatally faster to a maximal position at 2 years in the study. Patterns of weight growth were also similar. The values in achondroplasia were lower than those in the general population, but the difference was insignificant. The absolute values of growth parameters would vary due to ethnic differences. However, the growth patterns appeared to be similar because the genetic pathogenesis of the disease was shared across various populations.

### Influence of hydrocephalus and predictability of neurosurgical procedures

According to previous literature, hydrocephalus developed in 15–50% of patients with achondroplasia [7, 10, 15]. In our study, 15 of 125 patients were diagnosed with hydrocephalus, accounting for 12.0% of the cohort. The pathogenesis of hydrocephalus in achondroplasia was known to be related to increased pressure in the venous sinuses due to constriction of venous structures at the level of the jugular foramina and foramen magnum stenosis [5, 16, 24]. Therefore, for patients who presented with both hydrocephalus and craniocervical junction stenosis, FMD could be performed as an initial treatment. Previous studies described that hydrocephalus was relieved by FMD in achondroplasia [5, 16, 24]. Some patients who persistently presented hydrocephalus after FMD should undergo a VP shunt operation. It was known that both communicating and non-communicating hydrocephalus could be found in patients with achondroplasia. It has not been exactly known in which patients with hydrocephalus FMD worked [24]. Both types of hydrocephalus have the potential to be alleviated by FMD because FMD widens the craniocervical junction and makes redundant space for CSF flow. Increased venous pressure can be resolved by decompressing venous constriction.

To our knowledge, the influence of hydrocephalus on HC had never been evaluated in achondroplasia. Our study first evaluated changes in HC in achondroplasia according to the presence of hydrocephalus and whether FMD or VP shunt was performed or not. It showed no significant differences in HC between patients with and without hydrocephalus. There were no significant differences in HC between patients who required surgery (FMD or VP shunt) and those who did not. In previous literature, HC measurements had meaningful value for detecting hydrocephalus only for the first year of life in the general population [36, 37]. It was related to the compensatory expansion of the cranial vault before the fusion of cranial sutures. However, in achondroplasia, HC already considerably increased because of common ventriculomegaly and widened subarachnoid space. HC measurements had no meaningful value for detecting hydrocephalus, even in the first year of life.

Therefore, the diagnosis of hydrocephalus and the necessity of surgical treatments were not predictable using HC in achondroplasia. For treating hydrocephalus in time, detailed and thorough neurological evaluation with neuroimaging is essential. In our previous study [16], hydrocephalus was predictable using “frontal horn width”, “Evan’s ratio”, and “posterior indentation grade of the high cervical spinal cord”, which could be measured on MRI. Neuroimaging should be performed to evaluate both the brain and the craniocervical junction for patients at high risk of developing hydrocephalus and cervical myelopathy. Also, singular images could be insufficient for diagnosis because the ventricles remained enlarged in some cases. Identifying the increase in the ventricle size in the follow-up image can also be important.

### ETV as a treatment option for patients with hydrocephalus

There have been several works of literature that ETV was effective in treating achondroplasia patients with hydrocephalus [7, 14, 25, 26]. To our knowledge, it has been reported that 19 achondroplasia patients with hydrocephalus were treated with ETV. Although absolute indication was not established, patients who presented triventricular hydrocephalus and fourth ventricular outflow obstruction were successfully treated with ETV in the literature. Our 2 patients treated with ETV also presented triventricular hydrocephalus on MRI. The advantage of ETV is that there is no mechanical failure or infection of the device, as in the VP shunt. Furthermore, the malfunction rate of VP shunt is high in achondroplasia patients (48% in children at 5 years), and the recurrence rate of hydrocephalus after ETV is low in reported cases (the success rate at 6 months after surgery was reported to be 75.9%) [7, 14]. However, the steep angle of the third ventricular floor in achondroplasia patients often hinders a safe ETV procedure. Further study is needed to establish detailed surgical indications for ETV.

### Limitations

Our study had some limitations. First, this study was a retrospective analysis. The timing of measurement of height, weight, and head circumference was not constant among patients. Second, the number of patients with hydrocephalus and patients who underwent neurosurgical procedures was small. Further studies require more patients.

## Conclusion

The first Korean growth references for height, weight, and HC in young achondroplasia patients were constructed, including the growth patterns of patients with hydrocephalus and patients who underwent neurosurgical procedures such as FMD and VP shunt. The diagnosis of hydrocephalus and the timing of the neurosurgical procedures are not predictable using the HC of patients. Neuroimaging for evaluating the brain and the craniocervical junction is essential for achondroplasia patients who are at risk of developing hydrocephalus and cervical myelopathy.

## Data Availability

The datasets for the research presented in the current study are not publicly available due to individual privacy and lack of consent but available from the corresponding author upon reasonable request.
